# Immunotherapeutic Advancements for Glioblastoma

**DOI:** 10.3389/fonc.2015.00012

**Published:** 2015-01-29

**Authors:** Leonel Ampie, Eric C. Woolf, Christopher Dardis

**Affiliations:** ^1^Department of Neurology, St. Joseph’s Hospital and Medical Center, Barrow Neurological Institute, Phoenix, AZ, USA

**Keywords:** immunotherapy, glioblastoma, vaccines, antibodies, monoclonal, checkpoint modulators, T cell engineering

## Abstract

Immunotherapy seeks to improve the body’s immune response to a tumor. Currently, the principal mechanisms employed are: (1) to improve an aspect of the immune response (e.g., T cell activation) and (2) to encourage the targeting of particular antigens. The latter is typically achieved by exposing the immune system to the antigen in question, *in vivo*, or *in vitro* followed by re-introduction of the primed cells to the body. The clinical relevance of these approaches has already been demonstrated for solid tumors such as melanoma and prostate cancer. The central nervous system was previously thought to be immune privileged. However, we know now that the immune system is highly active in the brain and interacts with brain tumors. Thus, harnessing and exploiting this interaction represents an important approach for treating malignant brain tumors. We present a summary of progress in this area, focusing particularly on immune-checkpoint inhibition, vaccines, and T cell engineering.

## Introduction

Patients with cancer are typically immunosuppressed. This appears to be a survival strategy of the more aggressive tumors and is in excess of that which would be expected by external factors such as chemotherapy, malnutrition and steroid use. When discussing immunotherapy for tumors affecting the nervous system, the prototype remains glioblastoma (GB, grade IV glioma). This is the most common malignant primary central nervous system (CNS) malignancy (1). Aside from developments in the treatment of systemic metastases to the brain, the use of immunotherapy of other CNS tumors is at a relatively less developed stage.

An early observation germane to this field was that tumors may (rarely) resolve following an infection. This phenomenon has been documented, for example, in locally advanced pancreatic cancer (2). Therapeutic applications of this observation began with William Coley in 1891, when he injected inactivated *Streptococcus Pyogenes* and *Serratia Marcescen* into patients with sarcoma (3). By inducing systemic immune activation, it was hoped that the immune system would also increase its activity against the tumor. Indeed, the vaccine *did* cause tumor regression in some patients (4). Another relatively non-specific approach, which has proven to be of clinical value, has been the use of the Bacillus Calmette–Guérin (BCG) vaccine in those affected by bladder cancer (5).

These early, non-specific approaches suffered from unpredictable clinical responses. The use of genetically modified live bacteria remains under active investigation, principally *Salmonella* (6). In the case of GB, the addition of live bacteria to surgical wounds in the hopes of triggering local inflammation has proved controversial (7).

More tumor-specific therapies have been developed, which do not rely on a generalized immune response. Such approaches have already proven advantageous in highly immunogenic malignancies such as melanoma (4). Tumor-infiltrating lymphocytes are well recognized in GB. Studies to date have yielded conflicting data on the significance of these in relation to patient outcomes (8, 9). Nonetheless, their very presence makes enhancing their activity and specificity an attractive goal.

The gravity of GB has been a motivator for novel approaches. The median survival remains around 15 months and recurrence/progression is almost inevitable (10). Current treatment modalities include surgery, radiation, chemotherapy (temozolomide, bevacizumab, nitrosoureas), and electrical field treatment. This latter, known as NovoTTF-100A^®^, uses alternating electric fields to inhibit cell growth and has almost no side effects apart from local irritation of skin (11). The use of “targeted” chemotherapy, usually a single-agent specifically aimed at a particular cell-signaling pathway, has thus far been disappointing.

We focus on two emerging methods of harnessing the immune system in the treatment of GB:

- preventing the tumor from evading the immune system.
- exposing the immune system to antigens expressed by the tumor, thus stimulating it to attack the tumor.

To further illustrate these two points, we provide data from recently published clinical trials and from abstracts presented at the 2014 American Society of Clinical Oncology Annual Meeting (ASCO).

## CNS Immunology

The CNS was previously considered as a relatively ‘immune-privileged’ site. This was thought to reflect, in part, the protective nature of the blood–brain barrier (BBB). However, we now know that the CNS has an active and tightly regulated immune system (12). The circumventricular organs, which lack a BBB, have the ability to detect infection in the peripheral bloodstream. Areas with high vascularity, such as the leptomeninges and the choroid plexus, may also lead to microglial activation upon detection of pathogen-associated molecular patterns (PAMPs) in the bloodstream (13).

Microglia (phagocytic in function) are part of the evolutionarily older *innate* immune system. They are concentrated in the brain’s gray matter and are less numerous in white matter (the tracts of which may be used by GB to move to new locations) (14). Aside from the production of pro-inflammatory factors in the presence of infection, microglia are believed to play a role in removing neurotoxic debris (e.g., preventing the amyloid-β accumulation noted in Alzheimer’s disease).

The *adaptive* arm of the immune system (responsible for immunologic memory) was thought to be limited in the CNS due to the lack of lymphatic channels. Instead, cellular waste from the interstitial fluid is transferred to the CSF for removal via the glymphatic system. Circulating lymphocytes may be found within the CNS in their activated form but naïve T cells are essentially absent (15–17).

However infiltration of lymphocytes, especially T cells, is increased in patients harboring GB as the BBB becomes disrupted, suggesting an important interaction between the immune system and the tumor (18, 19). The tumor responds with a number of strategies to counteract the immune system. These include down regulation of major histocompatibility complex (MHC, responsible for presenting antigens) (20), an increase in cytotoxic T-lymphocyte-associated protein 4 (CTLA-4) and programed cell death protein 1 (PD-1) (21, 22), IL-10 (23), TGF-β (24), and by damping immune activity by recruiting regulatory T cells (T_Regs_) (25).

In addition to the BBB, the blood–tumor barrier must be overcome. The formation of new blood vessels by the tumor is often disorganized, with abnormal flow dynamics and immature pericytes, making recruitment of lymphocytes challenging. Experiments in mice and clinical observation support the view that immunotherapy is likely to be much less effective as the vasculature becomes more chaotic (26).

## Immune-Checkpoints

Immune-checkpoints prevent excessive immune activation, which may lead to collateral damage in healthy tissue. GB makes use of this apparatus to impair nearby T cell functionality. GB induces a state of chronic antigen exposure, which gradually increases the expression of immune-checkpoint proteins and culminates in lymphocytic exhaustion or anergy (27). By overcoming this habituation, it is hoped that immune-mediated cytotoxicity may be recovered.

While many proteins involved in this process have been identified, we focus here on two for which clinical applications have been developed: CTLA-4 and PD-1. Both are responsible for the down regulation of T cell activity (28). CTLA-4 is located on cytotoxic (CD8+) and the two major subsets of helper (CD4+) T cells. This protein restricts the activity of the T cell (29, 30). The ligand for CTLA-4 is similar to that of the co-stimulatory receptor CD28, (a complex of CD80 and CD86). It is thought to be a competitive agonist at this site (31, 32). T cell activation is inhibited by reducing both the production of IL-2 and the expression of its receptor, as well as arresting lymphocytes in the G1 phase of the cell cycle (33). Additionally, this immune-checkpoint protein has been shown to enhance the suppressive function of T_Reg_ cells (34, 35).

Ipilimumab is an antibody, which inactivates CTLA-4. This was the first agent focusing on immune-checkpoint blockade to receive approval from the FDA (36). It is used for patients with melanoma and has proven to be effective for those with brain metastases (37). In GB, a similar approach has been hampered by safety concerns. One review of 10 patients demonstrated that treatment was devoid of significant toxicities in all but 1 patient (38). However, in a subsequent study with five patients, all experienced auto-immune-related adverse effects (39). This typically consisted of a rash with colitis and hypothyroidism; there was also one case each of encephalitis and partial status epilepticus.

PD-1 expression is induced upon activation of a T cell; it serves to limit the potentially deleterious activity of lymphocytes in peripheral tissues. PD-1 has been shown to be expressed by T_regs_ and activation of its receptor appears to aid in their proliferation (40). PD-1 is also expressed by B cells and NK cells (41).

Nivolumab is a therapeutic antibody against PD-1. Is has proven to be effective when used with ipilimumab in patients with melanoma (42). There is an ongoing phase III trial comparing its efficacy with bevacizumab in patients with recurrent glioblastoma (NCT02017717). Pembrolizumab is another such antibody. Its activity in patients with metastatic melanoma depends on the presence of pre-existing cytotoxic T cells, which are thought to be deactivated by the tumor (43).

PD-1 binds to a ligand, PD-L1. This latter is up-regulated in numerous types of cancer (44). However, the use of PD-L1 as a biomarker for response to therapeutic checkpoint blockade is complicated by its heterogeneous expression in tumors, complex signaling networks, and the normal expression found on lymphocytes and other cells within the tumor microenvironment. In GB, expression of PD-L1 has been linked to the loss of the tumor suppressor PTEN (phosphatase and tensin homolog) and consequently the PI3K–Akt signaling pathway (phosphatidylinositol 3-kinase – protein kinase B a.k.a. Akt) (45). An antibody blocking PD-L1, MPDL3280A, has shown efficacy in the setting of metastatic bladder cancer in a phase I trial (46). This approach appears most effective in those patients in whom pre-existing immunity is suppressed by PD-L1, as evidenced by high levels of PD-L1 and CTLA-4 expression (47).

A more radical approach to recovery of immune function is that of bone-marrow transplant. Autologous progenitor cells have been used in GB to facilitate higher doses of cytotoxic chemotherapy. However, given the mortality with a complete marrow transplant, this has not been the subject of a trial. Experience with other tumor types suggests that this process “resets” the immune system and thus allows for recovery of cytotoxicity (48).

## Vaccines

Current approaches to immunotherapy may be classified as active or passive (49). “Passive” refers to antibodies to tumor antigens, or immune-conjugates aimed at targeted drug delivery (50). “Active” vaccines are intended to stimulate the patient’s own immune response. They may be cell-based (e.g., pulsed dendritic cells) or non-cell based (i.e., heat-shock protein-based vaccines).

### Peptide vaccines

Exposing short protein sequences to the immune system is usually done with peptides that are presented by HLA-A2 (human leukocyte antigen). This is the most common of the HLA subtypes but is found in only 50% of Caucasians and 30% of African-Americans. To overcome this limitation, antigens binding other class I HLAs have been developed, bringing population coverage to around 70%. Promising proteins from this line of investigation include: PTPRZ1 (receptor-type tyrosine-protein phosphatase zeta; function unclear but implicated in directional outgrowth of glioma cells), SEC61G (Protein transport protein Sec61 subunit gamma; involved in protein translocation across the endoplasmic reticulum for degradation), TNC (tenascin C; an extracellular glycoprotein typically expressed in development/differentiation and following injury), and EGFR (51).

EGFRvIII is a constitutively active mutant form of the epidermal growth factor receptor, which is present in approximately 33% of GB (52). Its presence is an independent negative prognostic indicator for survival in patients who manage to survive at least 1 year after initial diagnosis (53). A phase II trial was conducted in order to determine the immunogenicity, progression-free survival (PFS), and overall survival (OS) in patients who received a peptide-based vaccine (PEPvIII) targeted at EGFRvIII-expressing GB (54). Eligibility criteria included: gross total resection, Karnofsky performance status (KPS) ≥80%, and no evidence of progression after initial chemo-radiation. Immune reactivity after vaccination was monitored by observation of a delayed-type hypersensitivity (DTH) reaction to intradermal injections of PEPvIII and recall antigens. Eighteen patients were enrolled. Median PFS and OS were 14.2 and 26 months for those vaccinated vs. 6.3 and 15 months for controls. The skin test was performed in 17 patients; all showed no response prior to vaccination and all but 3 after vaccination. Of 14 patients tested, 6 demonstrated a positive humoral response against PEPvIII. The toxicity profile was deemed safe with most adverse reactions consisting of cutaneous reactions at the injection sites. (One patient had a severe allergic reaction). A phase III trial to confirm these results is ongoing.

### Heat-shock protein vaccines

Heat-shock proteins (HSP) are molecular chaperones; they provide protein stability by facilitating folding and aid in intra-cellular localization (55). Their activation is induced by adverse environments such as hypoxia, inflammation, and oxidative stress (56). Neoplastic cells are constantly exposed to such stressors; they rely on the HSP for survival.

A vaccine that includes HSP has proved safe and tolerable in a Phase I study of 12 patients with recurrent GB (57). After vaccination, peripheral leukocytes generally showed a response to HSP-96-bound peptides, as demonstrated by IFN-γ production (via real-time PCR). Lymphocytic infiltrates expressing IFN-γ were identified in those undergoing biopsy. Those showing an immune response to the vaccine showed an increase in median OS to 47 weeks vs. 16 in those with no response.

In the subsequent phase II trial, 41 patients with gross total resection of recurrent GB were vaccinated with HSPPC-96 (58). The median PFS of this cohort was 19.1 weeks with a median OS of 42.6 weeks. In both studies, the treatment appeared safe and tolerable.

### Autologous vaccines

These techniques rely on *ex vivo* modification of the patient’s immune system or of the tumor itself, followed by re-introduction of the altered cells. The immune system, particularly cytotoxic T lymphocytes, may be stimulated with tumor antigens. Neoplastic cells may be irradiated, or altered with viruses, in the hopes of increasing their immunogenicity and lowering their propensity for evasion of the immune system (49, 59).

Newcastle disease virus (NDV) combined with autologous tumor has been used as a vaccine. This virus has been shown to replicate selectively in neoplastic cells and to possess immunogenic properties (60). Twenty-three patients had their tumor surgically resected and incubated with hemagglutinating units of avirulent NDV. Concurrently, a control group was established, which comprised patients receiving standard care with a KPS of ≥60. An improvement in median PFS and OS was seen by comparison with controls: 40 weeks vs. 26 and 100 weeks vs. 49, respectively. Significant DTH skin reactions were noted when vaccinated patients were tested against irradiated tumor cells, both virus-modified and unmodified (61).

Autologous formalin-fixed tumor vaccines (AFTV) use fixed tissue to sensitize T cells to tumor antigens. In a Phase I/IIa trial, 22 newly diagnosed patients with resected GB received AFTV with concomitant fractionated radiotherapy (62–65). Median PFS and OS were promising at 7.6 and 19.8 months. Again, the treatment combination was well tolerated and adverse events were mostly limited to cutaneous reactions induced by the injection (66).

### Dendritic-cell-based vaccines

This process involves obtaining dendritic cells from a patient and pulsing them with glioma antigens derived from a resection. A major advantage is that multiple antigens may thus be presented (49, 67). This is of particular relevance to GB, which is known to display high intra-tumoral heterogeneity. Evidence of efficacy has already been established for metastatic prostate cancer with sipuleucel-T, although those with nervous system metastases were excluded from the pivotal trials (68).

DCVax-L^®^ is another such dendritic-cell-based vaccine. In a phase I clinical trial, 23 patients with resected GB had an immunogenic lysate prepared from their tumor plus dendritic-cells derived from peripheral blood mononuclear cells (PBMC). The dendritic cells were supplemented with granulocyte-macrophage colony-stimulating factor (GM-CSF) and IL-4 before exposure to the lysate. The treatment was safe, tolerable, and without evidence of dose-limiting toxicity (69). The median PFS and OS were 15.9 and 31.4 months, respectively. A randomized phase III trial is ongoing (NCT00045968).

This approach is also being explored as a way to target glioma stem cells, which represent a radioresistant and chemoresistant subpopulation of cells within a patient’s tumor. In a phase I trial, 17 patients with newly diagnosed GB were given a dendritic-cell-based vaccine with a combination of glioma stem cell antigens. This approach (the ICT-107 vaccine) reported a promising median PFS and OS of 16.9 and 38.4 months, respectively. Interestingly, five patients who underwent a subsequent resection had a decrease or absence of cells positive for CD133, a glioma stem cell marker (70). A phase II trial was initiated with the same vaccine but despite currently unpublished data demonstrating a significant increase in PFS, there was no increase in OS (49). A phase III trial is planned nonetheless. A similar concept has been applied in the production of a vaccine (ICT-121) that targets CD133-positive glioma cells (CD 133 is an enrichment marker for cancer stem cells). A phase I trial involving this vaccine is underway (NCT02049489).

### Viral protein-based vaccines

A variety of studies have identified human cytomegalovirus (CMV) proteins and nucleic acids in approximately 90–100% of primary GBs (71–73). Although the role of CMV in the pathogenesis and progression of GB is not fully understood, the prevalence of these antigens in tumor cells and relative absence in normal surrounding tissue provides an important opportunity to develop targeted immunotherapeutics (74). Interestingly, one patient receiving DCVax-L developed a specific anti-CMV (anti-pp65) cytotoxic T cell response (75).

To date, immunotherapeutic targeting of CMV has been tried in a limited number of patients with high-grade gliomas. One case study describes a patient with recurrent GB who received adoptive transfer of CMV-specific T cells concurrently with temozolomide, which resulted in 17 months without disease progression (76). Recently, a trial involving patients with GB demonstrated that the transfer of expanded CMV-specific T cells lead to a median OS of 403 days (vs. historical median OS of 180 days) and 4/10 patients who completed the treatment remained progression-free during the study period (77). Ongoing trials are assessing the use of CMV-specific dendritic-cell vaccines (NCT00639639) and CMV-specific T cells following drug-induced lymphopenia in GB (NCT00693095). Direct targeting of CMV with valganciclovir has been the subject of some controversy and is not currently recommended outside the context of a clinical trial (78).

## T Cell Engineering

Adoptive cell transfer using genetically engineered T cells represents another attractive immunotherapeutic approach to treating GB. T cells that recognize specific tumor-associated antigens (TAAs) can be generated by fusing an extracellular binding domain (usually derived from a TAA-specific monoclonal antibody) to the intra-cellular signaling domain of the T cell receptor (TCR) to form a chimeric antigen receptor (CAR) (79). CAR T cell activation is MHC-independent and therefore circumvents issues involving down regulation of HLA class I molecules and defects in antigen processing that tumors use to evade T cell recognition (80). These engineered cells are also potentially more useful than antibody-based immunotherapies because they have the ability to migrate through blood vessel walls, penetrate solid tumor, and recruit addition components of the immune response (81). CARs have been developed for glioma-specific antigens, including HER2, IL-13Rα2, and EGFRvIII, and have demonstrated potent antitumor activity with *in vivo* models (81, 82).

Interestingly, the CARs generated against HER2 in GB patients, also recognized the CD133+ stem cell populations, that are thought to contribute to tumor recurrence (80). Mounting evidence that this has led to a number of clinical trials exploring the safety and effectiveness of CARs against HER2 (NCT01109095), IL-13Rα2 (NCT00730613; NCT01082926), and EGFRvIII (NCT01454596).

## What have We Learned?

Although immunotherapy has been with us for over a century, we are still in the preliminary stages of refining this therapeutic approach. Thus far, immune-based treatments have proven to be relatively safe with minimal toxicities, especially by comparison with traditional cytotoxic chemotherapy. Currently, it is estimated that <20% of patients with GB enroll in clinical trials, so increasing participation would appear to be a clear priority. Given the variety of methods receiving attention, much of the field is anticipated to be in phase I and II trials for some years (Table 1). Hence, the usual caveats apply regarding lack of power, lack of randomization, and the use of historical controls. In spite of this, the preliminary survival data have, on the whole, been encouraging.

**Table 1:** **Immunotherapy-based clinical trials for glioblastoma, which are currently recruiting**.

Trial name	Phase	Target accrual	Therapy	Primary outcome	Identifier
**PEPTIDE-BASED**					
Phase I/II trial of IMA950 multi-peptide vaccine plus poly-ICLC in glioblastoma	I/II	16	IMA950 multipeptide based vaccine/poly-ICLC/temozolomide/radiotherapy	Safety, tolerability	NCT01920191
Safety and efficacy study of SL-701, a glioma-associated antigen vaccine to treat recurrent glioblastoma multiforme	I/II	100	SL-701/imiquimod cream 5%/sargramostim 150 mg	Safety, tolerability, OS, ORR	NCT02078648
GAPVAC Phase I trial in newly diagnosed glioblastoma patients	I	20	APVAC1 vaccine/poly-ICLC/GM-CSF	Safety, feasibility, biological activity	NCT02149225
			APVAC2 vaccine/poly-ICLC/GM-CSF	
Phase I study of safety and immunogenicity of ADU-623	I	38	ADU-623	Safety, tolerability, immunogenicity	NCT01967758
**IMMUNE CHECKPOINT BASED**					
A randomized study of nivolumab vs. bevacizumab and a safety study of nivolumab in adult subjects with recurrent glioblastoma (GBM) (CheckMate 143)	III	260	Nivolumab, bevacizumab, ipilimumab	Safety, tolerability, efficacy	NCT02017717
**HEAT-SHOCK PROTEIN BASED**					
Research for immunotherapy of glioblastoma with autologous heat-shock protein gp96	I	20	gp96	Safety, efficacy	NCT02122822
**AUTOLOGOUS-BASED**					
Randomized phase II multicentre study to investigate efficacy of autologous lymphoid effector cells specific against tumor-cells (ALECSAT) in patients with glioblastoma multiform measured compared to avastin/irinotecan	II	175	ALECSAT/bevacizumab/irinotecan	PFS	NCT02060955
Pilot study of autologous t cells redirected to EGFRVIII-With a chimeric antigen receptor in patients with EGFRVIII + glioblastoma	I	12	CART-EGFRvIII T cells	Safety, feasibility	NCT02209376
**DENDRITIC-CELL BASED**					
Study of a drug [DCVax^®^-L] to treat newly diagnosed GBM brain cancer	III	300	DCVax^®^-L	Efficacy, PFS	NCT00045968
A study of ICT-121 dendritic cell vaccine in recurrent glioblastoma	I	20	ICT-121 DC vaccine	Safety, tolerability	NCT02049489
Phase I study of a dendritic cell vaccine for patients with either newly or recurrent glioblastoma	I	40	^a^Dendritic cell vaccination/temozolomide/radiotherapy	Safety, tolerability	NCT02010606
			^a^Dendritic cell vaccination ± bevacizumab (for patients previously treated with bevacizumab)	
Dendritic cell vaccine for patients with brain tumors	II	60	Autologous tumor lysate-pulsed DC vaccination ± (0.2% resiquimod or adjuvant poly-ICLC)	Efficacy	NCT01204684
Basiliximab in treating patients with newly diagnosed glioblastoma multiforme undergoing targeted immunotherapy and temozolomide-caused lymphopenia (REGULATe)	I	18	RNA-loaded dendritic cell vaccine (basiliximab)	Safety, efficacy	NCT00626483
Vaccine therapy with or without sirolimus in treating patients with NY-ESO-1 expressing solid tumors	I	30	DEC-205-NY-ESO-1 ± sirolimus	Safety, tolerability	NCT01522820 *(not glioma-specific)*
Ph I personalized neoantigen cancer vaccine with radiotherapy for patients with MGMT unmethylated, newly diagnosed glioblastoma	I	20	Radiotherapy, personalized NeoAntigen Vaccine (NeoVax)	Safety, efficacy	NCT02287428
Dendritic cell vaccine for malignant glioma and glioblastoma multiforme in adult and pediatric subjects	I	20	DC vaccination/tumor lysate/imiquimod	Safety, efficacy	NCT01808820
Vaccine therapy and temozolomide in treating patients with newly diagnosed glioblastoma	I	10	DC vaccination/temozolomide	Safety	NCT01957956
Dendritic cell vaccine therapy with *in situ* maturation in pediatric brain tumors	I	20	DC vaccination/tumor lysate, imiquimod	Safety	NCT01902771
**T-CELL BASED THERAPY**					
CAR T cell receptor immunotherapy targeting EGFRvIII for patients with malignant gliomas expressing EGFRvIII	I/II	160	Anti-EGFRvIII CAR transduced PBL/aldesleukin/fludarabine/cyclophosphamide	Safety, PFS	NCT01454596
Cellular immunotherapy study for brain cancer (alloCTL)	I	15	Alloreactive CTL	Safety, efficacy	NCT01144247
CMV-specific cytotoxic T lymphocytes expressing CAR targeting HER2 in patients with GBM (HERT–GBM)	I	18	HER2.CAR CMV-specific CTLs	Safety	NCT01109095

*Therapy: Poly ICLC, an immunostimulant and ligand for the toll-like receptor; composed of carboxymethylcellulose, polyInosinic-polyCytidylic acid, and poly-l-lysine double-stranded RNA; Sargramostim, recombinant granulocyte–monocyte colony-stimulating factor; GM-CSF, granulocyte–monocyte colony-stimulating factor; APVAC, activated personalized vaccination; DC, dendritic cell; PBL, peripheral blood lymphocytes; CAR, chimeric antigen receptor; Aldesleukin, recombinant IL-2; CMV, cytomegalovirus; CTL, cytotoxic T lymphocyte*.

*Outcomes: OS, overall survival; PFS, progression free survival; ORR, objective response rate*.

*Retrieved from https://clinicaltrials.gov/ on 12/18/2014*.

Using peripheral immune reactivity as a surrogate marker for disease activity (and thus outcomes) is attractive, in that it may allow for more rapid development of active agents. In practice, it has thus far led to mixed results. While some trials link immune reactivity with a better prognosis, others show no such association (83). It is hoped that greater standardization and more refined methods will overcome these difficulties.

Trials to date have studied the effects of immune-checkpoint inhibitors and vaccines separately. As our knowledge of these treatments increases, we can begin to consider combining both. Such an approach has already been shown to be efficacious in a murine model of glioma (84).

Approaches targeting specifically just one antigen have the drawback that evolution of resistance appears almost inevitable in those with GB. Such difficulties are well recognized in solid tumors to which “targeted” approaches have been applied: at least two such agents are thought to be necessary (to inhibit tumor growth) and preferably three (85). Those which aim to simulate the immune system or expose it to a broad range of antigens thus hold greater promise. As data on the safety of single-agent approaches accrues and as patents expire, rational multi-agent combinations are likely to become the norm for most patients.

## Conflict of Interest Statement

The authors declare that the research was conducted in the absence of any commercial or financial relationships that could be construed as a potential conflict of interest.
